# Characteristics of new users of recent antidiabetic drugs in Canada and the United Kingdom

**DOI:** 10.1186/s12902-022-01140-1

**Published:** 2022-09-29

**Authors:** Vanessa C. Brunetti, Audray St-Jean, Sophie Dell’Aniello, Anat Fisher, Oriana H. Y. Yu, Shawn C. Bugden, Jean-Marc Daigle, Nianping Hu, Silvia Alessi-Severini, Baiju R. Shah, Paul E. Ronksley, Lisa M. Lix, Pierre Ernst, Kristian B. Filion, for the Canadian Network for Observational Drug Effect Studies (CNODES) Investigators, Samy Suissa, Colin R. Dormuth, Brenda R. Hemmelgarn, Jacqueline Quail, Dan Chateau, J. Michael Paterson, Jacques LeLorier, Adrian R. Levy, Pierre Ernst, Kristian B. Filion, Robert W. Platt, Ingrid S. Sketris

**Affiliations:** 1 Department of Epidemiology, Biostatistics, and Occupational Health, McGill University, Montreal, Quebec Canada; 2 Center for Clinical Epidemiology, Lady Davis Institute, Jewish General Hospital, 3755 Côte Ste Catherine, Suite H410.1, Montreal, Quebec H3T 1E2 Canada; 3 Department of Anesthesiology, Pharmacology and Therapeutics, University of British Columbia, Vancouver, British Columbia Canada; 4 Division of Endocrinology, Department of Medicine, Jewish General Hospital, Quebec, Montreal Canada; 5 College of Pharmacy, Rady Faculty of Health Sciences, University of Manitoba, Winnipeg, Manitoba Canada; 6 School of Pharmacy, Memorial University of Newfoundland, St John’s, Newfoundland and Labrador Canada; 7 Institut national d’excellence en santé et en services sociaux (INESSS), Quebec, Quebec Canada; 8 The Health Quality Council, Saskatoon, Saskatchewan Canada; 9 Manitoba Centre for Health Policy, Rady Faculty of Health Sciences, University of Manitoba, Winnipeg, Manitoba Canada; 10 ICES, Toronto, Ontario Canada; 11 Sunnybrook Health Sciences Centre, Toronto, Ontario Canada; 12 Department of Community Health Sciences, Cumming School of Medicine, University of Calgary, Calgary, Alberta Canada; 13 Department of Community Health Sciences, University of Manitoba, Winnipeg, Manitoba Canada; 14 Department of Medicine, McGill University, Quebec, Montreal Canada

**Keywords:** Type 2 diabetes, sodium-glucose co-transporter 2 inhibitors, glucagon-like peptide 1 receptor agonists, dipeptidyl peptidase 4 inhibitors

## Abstract

**Background:**

Characteristics of patients using newer 2^nd^ and 3^rd^ line antidiabetic drugs in a real-world setting are poorly understood. We described the characteristics of new users of sodium-glucose co-transporter-2 inhibitors (SGLT-2i), dipeptidyl peptidase-4 inhibitors (DPP-4i), and glucagon-like peptide-1 receptor agonists (GLP-1 RA) in Canada and the United Kingdom (UK) between 2016 and 2018.

**Methods:**

We conducted a multi-database cohort study using administrative health databases from 7 Canadian provinces and the UK Clinical Practice Research Datalink. We assembled a base cohort of antidiabetic drug users between 2006 and 2018, from which we constructed 3 cohorts of new users of SGLT-2i, DPP-4i, and GLP-1 RA between 2016 and 2018.

**Results:**

Our cohorts included 194,070 new users of DPP-4i, 166,722 new users of SGLT-2i, and 27,719 new users of GLP-1 RA. New users of GLP-1 RA were more likely to be younger (mean ± SD: 56.7 ± 12.2 years) than new users of DPP-4i (67.8 ± 12.3 years) or SGLT-2i (64.4 ± 11.1 years). In Canada, new users of DPP-4i were more likely to have a history of coronary artery disease (22%) than new users of SGLT-2i (20%) or GLP-1 RA (15%).

**Conclusion:**

Although SGLT-2i, DPP-4i, and GLP-1 RAs are recommended as 2^nd^ or 3^rd^ line therapy for type 2 diabetes, important differences exist in the characteristics of users of these drugs. Contrary to existing guidelines, new users of DPP-4i had a higher prevalence of cardiovascular disease at baseline than new users of SGLT2i or GLP-1RA.

**Supplementary Information:**

The online version contains supplementary material available at 10.1186/s12902-022-01140-1.

## Introduction

Several new classes of drugs used to manage type 2 diabetes have entered the market over the last two decades, including dipeptidyl peptidase-4 inhibitors (DPP-4i), sodium-glucose co-transporter-2 inhibitors (SGLT-2i), and glucagon-like peptide-1 receptor agonists (GLP-1 RAs). American, United Kingdom (UK), and Canadian guidelines for the treatment of type 2 diabetes all recommend using these therapies as replacement or in addition to first-line treatment with metformin if target glycemic levels are not reached [1–4]. Because of additional beneficial effects, some of these drugs may be preferred over others in specific sub-populations. Randomized controlled trials (RCTs) suggest that GLP-1 RAs [5, 6] and SGLT-2i [7, 8] may also have cardioprotective and weight-loss effects, which may be particularly beneficial in some sub-population, such as patients at higher risk of cardiovascular outcomes [9, 10]. In addition, SGLT-2i have been shown to have beneficial effects on heart failure in patients with type 2 diabetes [11]. They also have benefits on renal outcomes [12] and are recommended for patients with type 2 diabetes and chronic kidney disease [2]. With the growing availability and diversity of antidiabetic treatments, it is essential to understand the characteristics of the patients using them, which remain unclear to date. The objective of this study was to describe the characteristics of new users of DPP-4i, SGLT-2i, and GLP-1 RA in Canada and in the UK between 2016 and 2018.

## Methods

### Data sources

This study was conducted by the Canadian Network for Observational Drug Effect Studies (CNODES) [13]. We conducted a multi-database cohort study using linkable administrative health databases from 7 Canadian provinces (Alberta, British Columbia, Manitoba, Nova Scotia, Ontario, Quebec, and Saskatchewan) and the UK Clinical Practice Research Datalink (CPRD) Gold [14]. A common protocol was implemented in each participating site. The Canadian databases contain population-level data on physician claims, hospitalization records, and prescription drugs dispensed from community pharmacies (Table S1). The CPRD is a primary care database that contains the full general practitioner records of over 15 million patients in over 700 practices in the UK^15^. Prescription rates of antidiabetic drugs are similar in the CPRD Gold and CPRD Aurum [15]. CPRD data were linked to the Hospital Episode Statistics [14] database, which contains data on hospitalization records, and to the Office for National Statistics database, which contains data on vital status. Linkage is available for general practices in England that have consented to the linkage scheme (currently representing 75% of all English practices).

### Study population

In each jurisdiction, we identified a base cohort that included all patients who were dispensed (in the Canadian databases) or prescribed (in the CPRD) an antidiabetic drug between January 1, 2006 and June 30, 2018 (or the latest date of data availability at each site; Table S1). Antidiabetic drugs included metformin, sulfonylureas, thiazolidinediones, DPP-4i, SGLT-2i, GLP-1 RA, alpha-glucosidase inhibitors, meglitinides, insulin, or a combination of these drugs. DPP-4i were approved in Canada and the UK in 2007; a base cohort that began in 2006 allowed for the inclusion of the entire period during which the newer antidiabetic drugs (DPP-4i, SGLT-2i, GLP-1 RA) had regulatory approval in all included jurisdictions. The date of the first dispensing (Canadian databases) or prescription (CPRD) defined entry into the base cohort. From this base cohort, we then created separate cohorts for each of the three study drugs. These cohorts were restricted to new users of DPP-4i, SGLT-2i, and GLP-1 RAs who received their first dispensing between January 1, 2016 and June 30, 2018 (or latest date of data availability in each site). We restricted the study period to 2016 to 2018 to reflect contemporary use of these drugs. We defined new use as a first dispensing or prescription for each drug class with no use in the preceding year except in Quebec, where new use was defined as no prior use at any time. Due to prescription drug data availability, inclusion was restricted to those aged ≥19 years in Alberta, those aged ≥66 years in Ontario, and those aged ≥66 years, social assistance recipients, and those without access to a private prescription drug insurance plan in Quebec. In Nova Scotia, inclusion was restricted to November 1, 2017 to June 30, 2018 due to the limited availability of prescription drug data. As the Ontario public drug plan did not cover GLP-1 RAs during the study period, we were unable to report data on their use in this province. The provincial formulary listings for the three classes of study drugs during the study period are summarized in Table 1 [16]. Ontario and Quebec only include dispensations under the provincial drug programs, whereas other provinces include any dispensations regardless of payer. In all sites, patients were permitted to contribute to ≥1 study cohort.

**Table 1:** Formulary listings for DPP-4 inhibitors, SGLT-2 inhibitors, and GLP-1 receptor agonists in the Canadian provinces in October 2018

		AB	BC	MB	NS	ON	QC	SK
**DPP-4 inhibitors**	Alogliptin	NL	NL	NL	NL	NL	R	NL
	Alogliptin + metformin	NL	NL	NL	NL	NL	R	NL
	Linagliptin	R	R	R	R	L	R	R
	Linagliptin + metformin	R	R	R	R	L	R	R
	Sitagliptin	R	DL	R	R	L	R	R
	Sitagliptin+ metformin	R	DL	R	R	L	R	R
	Sitagliptin + ertugliflozin	NL	NL	NL	NL	NL	NL	NL
	Saxagliptin	R	R	R	R	L	R	R
	Saxagliptin + metformin	R	R	R	R	L	R	R
**SGLT-2 inhibitors**	Canagliflozin	R	NL	R	R	L	R	R
	Canagliflozin + metformin	NL	NL	NL	NL	NL	NL	NL
	Dapagliflozin	R	NL	R	R	L	R	R
	Dapagliflozin + metformin	R	NL	R	R	L	R	R
	Empagliflozin	R	NL	R	R	R	R	R
	Empagliflozin + metformin	NL	NL	NL	NL	NL	R	NL
**GLP-1 receptor agonists**	Albiglutide	NL	NL	NL	NL	NL	NL	NL
	Dulaglutide	NL	NL	NL	NL	NL	NL	NL
	Exenatide	NL	NL	NL	NL	NL	NL	NL
	Liraglutide	NL	NL	NL	NL	NL	R	NL
	Liraglutide + insulin degludec	NL	NL	NL	NL	NL	NL	NL
	Lixisenatide	NL	NL	NL	NL	NL	R	NL
	Lixisenatide + insulin glargine	NL	NL	NL	NL	NL	NL	NL
	Semaglutide	NL	NL	NL	NL	NL	NL	NL

*Abbreviations*: *AB* Alberta, *BC* British Columbia, *MB* Manitoba, *NS* Nova Scotia, *ON* Ontario, *QC* Quebec, *SK* Saskatchewan, *NL* Not listed – not available through the public drug plan, *R* Restricted – only available to those who meet eligibility criteria and receive prior approval from the drug benefit plan, cost will be fully or partially covered according to the terms of the public drug plan; *L* Listed – Can be prescribed by any doctor, cost will be fully or partially covered according to the terms of the public drug plan; *DL* Delisted – product has been removed from the formulary and is no longer available

### Patient characteristics

Patient characteristics, including demographic information (age, sex, income quintile) and diabetes duration (time since first diabetes diagnosis or treatment), were assessed at cohort entry. Comorbidities (history of myocardial infarction, ischemic stroke, diabetic ketoacidosis, retinopathy, neuropathy, nephropathy, cancer, dialysis, other kidney diseases, alcohol related disorders, cirrhosis, atrial fibrillation, chronic obstructive pulmonary disease, coronary artery disease, dyslipidemia, hypertension, heart failure, venous thromboembolism, peripheral arterial disease, aortic aneurysm, atherosclerosis, cerebrovascular disease, pyelonephritis, cystitis) were assessed in the 3 years prior to cohort entry (Table S2). Prior medication use (metformin, sulfonylureas, thiazolidinediones, DPP-4i, SGLT-2i, GLP-1 RA, alpha-glucosidase inhibitors, meglitinides, insulin, angiotensin-converting enzyme inhibitors, angiotensin receptor-blockers, beta-blockers, calcium channel blockers, loop diuretics, thiazide diuretics, other diuretics, direct renin inhibitors, aldosterone antagonists, digitalis-like agents, statins, other lipid lowering therapy, acetylsalicylic acid, non-acetylsalicylic acid antiplatelets, nonsteroidal anti-inflammatory drugs, oral anticoagulants, oral glucocorticoids, atypical antipsychotics) and health care use (number of inpatient hospitalizations, number of physician visits) were assessed in the 1 year prior to cohort entry (Table S2). We also assessed current use of medications on the date of study cohort entry. Comorbidities were assessed using the 8^th^ (for Ontario physician claims only), 9^th^, and 10^th^ revisions of the International Classification of Diseases and Related Health Problems with Canadian Enhancement (ICD-9-CM and ICD-10-CA) in physician claims and hospitalization records in the 3 years before study cohort entry (Table S2). In the CPRD, comorbidities were assessed using ICD-10 and Read codes (clinical terminology in the UK [17]). In addition, the following characteristics available only in the CPRD were assessed: body mass index (BMI), smoking status, race, blood pressure, glycated hemoglobin (HbA1c), and estimated glomerular filtration rate (eGFR) using the most recent measurement at any time prior to cohort entry.

### Statistical analyses

Descriptive statistics were used to describe the demographic and clinical characteristics of new users of DPP-4i, SGLT-2i, and GLP-1 RA at each site. Discrete data were described using counts and percentages; continuous data were described using means and standard deviations (SD). Counts were summed across sites and continuous variables were pooled across sites using weighted means and SDs. We also conducted qualitative, descriptive comparisons between the Canadian sites and the UK site. Due to differences in prescription drug data available across Canadian sites (i.e., publicly vs. privately reimbursed claims), we conducted a sensitivity analysis to explore whether these differences could be a source of between-site heterogeneity. Using Manitoba data (a province with information on reimbursement status), we compared the characteristics of patients using DPP-4i and SGLT-2i by reimbursement status. GLP-1 RA were not considered in this analysis, as these drugs were not covered by the provincial health plan in Manitoba during the study period. All analyses were performed using SAS (versions varied across sites).

## Results

There were 2,175,815 users of antidiabetic drugs between 2016 and 2018. When data were pooled across jurisdictions, our study cohort included 194,070 new users of DPP-4i, 166,722 new users of SGLT-2i, and 27,719 new users of GLP-1 RA. Tables 2, 3 and 4 summarize the characteristics of new users of these treatments at each participating site. In general, the proportion of new users of DPP-4i and SGLT-2i was similar across study sites, with fewer patients initiating GLP-1 RA. Overall, new users of GLP-1 RA were younger than new users of DPP-4i or SGLT-2i. New users of GLP-1 RAs were also less likely to be male than new users of DPP-4i or SGLT-2i, except in Quebec and the UK. Mean duration of diabetes was similar for new users of DPP-4i and SGLT-2i across sites. However, differences in the duration of diabetes were observed among new users of GLP-1 RAs; approximately 20% of patients had <1 year of history of diabetes in all sites except in Quebec and in the UK, where <3% had a diabetes duration of <1 year.

In Canada, between 27.6% and 44.5% of new users of GLP-1 RAs used ≥3 antidiabetic medications in the year prior to initiation in all provinces except Quebec, where 81.0% used ≥3 antidiabetic medications in the prior year (Table 3). The majority of users in all three groups used metformin prior to initiating treatment with the study drugs (range: 61.7 – 89.4%) (Tables 2, 3, and 4). In addition, 16.2%, 25.9%, and 26.9% of new users in Canada had used insulin prior to initiating treatment with DPP-4i, SGLT-2i, and GLP-1 RA, respectively (Table S4). Concurrent antidiabetic drug use was generally similar across groups.

**Table 2:** Characteristics of new users of DPP-4 inhibitors by study site, 2016-2018

	AB (***n*** = 12,874)	BC (***n*** = 24,388)	MB (***n*** = 5,605)	NS (***n*** = 1,049)	O***N*** (***n*** = 67,484)	QC (***n*** = 64,332)	SK (***n*** = 13,148)	UK (***n*** = 5,190)
**Age (years)**	59.9 **±** 12.7	63.0 **±** 12.8	58.5 **±** 13.5	58.9 **±** 11.6	74.9 **±** 7.1	66.3 **±** 12.0	61.2 **±** 13.3	65.1 **±** 13.6
< 18	**–**	8 (0.0)	s	0 (0.0)	**–**	6 (0.0)	8 (0.1)	s
18-35	403 (3.1)	487 (2.0)	275 (4.9)	29 (2.8)	**–**	942 (1.5)	374 (2.8)	s
36-45	1,452 (11.3)	1,697 (7.0)	675 (12.0)	87 (8.3)	**–**	2,613 (4.1)	1,211 (9.2)	323 (6.2)
46-55	3,038 (23.6)	4,463 (18.3)	1,325 (23.6)	273 (26.0)	**–**	7,082 (11.0)	2,713 (20.6)	919 (17.7)
56-65	3,854 (29.9)	7,098 (29.1)	1,605 (28.6)	393 (37.5)	**–**	18,589 (28.9)	3,830 (29.1)	1,230 (23.7)
66-75	2,737 (21.3)	6,601 (27.1)	1,126 (20.1)	174 (16.6)	40,155 (59.5)	21,545 (33.5)	3,154 (24.0)	1,370 (26.4)
76-85	1,090 (8.5)	3,228 (13.2)	480 (8.6)	82 (7.8)	20,901 (31.0)	10,412 (16.2)	1,414 (10.8)	952 8.3)
> 85	300 (2.3)	806 (3.3)	s	11 (1.1)	6,428 (9.5)	3,143 (4.9)	444 (3.4)	314 (6.1)
**Females**	5,219 (40.5)	10,315 (42.3)	2,686 (47.9)	471 (44.9)	31,560 (46.8)	27,803 (43.2)	5,679 (43.2)	2,234 (43.0)
**Income quintile** ^a^								
1^st^ (lowest)	3,321 (25.8)	8,555 (35.1)	1,503 (26.8)	153 (14.6)	16,419 (24.3)	6,871 (10.7)	3,156 (24.0)	1,688 (32.5)
2^nd^	2,850 (22.1)	**–**	1,265 (22.6)	185 (17.6)	15,175 (22.5)	**–**	2,460 (18.7)	1,056 (20.3)
3^rd^	2,315 (18.0)	**–**	1,066 (19.0)	170 (16.2)	13,589 (20.1)	**–**	2,838 (21.6)	950 (18.3)
4^th^	2,085 (16.2)	**–**	945 (16.9)	188 (17.9)	11,845 (17.6)	**–**	2,541 (19.3)	938 (18.1)
5^th^ (highest)	1,786 (13.9)	**–**	781 (13.9)	133 (12.7)	10,311 (15.3)	–	2,087 (15.9)	558 (10.8)
Missing	517 (4.0)	**–**	45 (0.8)	220 (21.0)	145 (0.2)	–	66 (0.5)	0 (0.0)
**Calendar year at cohort entry**								
2016	10,311 (80.1)	9,367 (38.4)	2,566 (45.8)	**–**	30,564 (45.3)	25,875 (40.2)	9,793 (74.5)	3,041 (58.6)
2017	2,563 (19.9)	9,922 (40.7)	2,416 (43.1)	238 (22.7)	29,905 (44.3)	25,399 (39.5)	2,228 (17.0)	2,149 (41.4)
2018	0 (0.0)	5,099 (20.9)	623 (11.1)	811 (77.3)	7,015 (10.4)	13,058 (20.3)	1,127 (8.6)	0 (0.0)
**Diabetes duration (years)**	9.4 **±** 6.3	11.2 **±** 7.6	11.4 **±** 7.9	8.6 **±** 6.4	13.1 **±** 7.6	10.8 **±** 6.9	11.3 **±** 6.9	10.4 **±** 7.2
< 1 year	992 (7.7)	2,884 (11.8)	412 (7.4)	138 (13.2)	4,988 (7.4)	5,834 (9.1)	667 (5.1)	306 (5.9)
1-4.9 years	2,881 (22.4)	3,200 (13.1)	1,004 (17.9)	236 (22.5)	7,044 (10.4)	9,803 (15.2)	2,087 (15.9)	1,010 (19.5)
5-10 years	3,471 (27.0)	5,156 (21.1)	1,326 (23.7)	268 (25.6)	12,268 (18.2)	14,356 (22.3)	3,372 (25.7)	1,405 (27.1)
> 10 years	5,530 (43.0)	13,148 (53.9)	2,863 (51.1)	407 (38.8)	43,184 (64.0)	34,339 (53.4)	7,022 (53.4)	2,469 (47.6)
**Use of medications** ^‡^								
No. of antidiabetic drugs	1.6 **±** 1.0	1.6 **±** 1.0	1.9 **±** 0.8	1.0 **±** 1.0	1.3 **±** 0.8	1.4 **±** 0.9	2.5 **±** 1.0	1.4 **±** 0.9
0	1,405 (10.9)	3,804 (15.6)	338 (6.0)	128 (12.2)	10,676 (15.8)	10,249 (15.9)	431 (3.3)	728 (14.0)
1	4,589 (35.7)	7,345 (30.1)	1,227 (21.9)	436 (41.6)	31,842 (47.2)	22,625 (35.2)	1,361 (10.4)	2,286 (44.0)
2	4,877 (37.9)	9,583 (39.3)	2,986 (53.3)	386 (36.8)	20,266 (30.0)	26,583 (41.3)	4,782 (36.4)	1,757 (33.9)
≥ 3	2,003 (15.6)	3,656 (15.0)	1,054 (18.8)	99 (9.4)	4,700 (7.0)	4,875 (7.6)	6,574 (50.0)	419 (8.1)
Prior antidiabetic drugs								
Metformin	10,371 (80.6)	18,155 (74.4)	4,730 (84.4)	811 (77.3)	48,983 (72.6)	48,206 (74.9)	11,448 (87.1)	4,042 (77.9)
Sulfonylureas	4,796 (37.3)	11,330 (46.5)	3,925 (70.0)	425 (40.5)	20,028 (29.7)	28,757 (44.7)	8,180 (62.2)	1,900 (36.6)
Thiazolidinediones	444 (3.5)	331 (1.4)	140 (2.5)	7 (0.7)	503 (0.7)	952 (1.5)	390 (3.0)	261 (5.0)
SGLT2 inhibitors	1,431 (11.1)	3,756 (15.4)	766 (13.7)	112 (10.7)	4,632 (6.9)	2,566 (4.0)	1,769 (13.5)	349 (6.7)
GLP-1 receptor agonists	369 (2.9)	582 (2.4)	26 (0.5)	33 (3.2)	0 (0.0)	111 (0.2)	92 (0.7)	116 (2.2)
Alpha-glucosidase inhibitors	43 (0.3)	241 (1.0)	89 (1.6)	s	397 (0.6)	327 (0.5)	68 (0.5)	12 (0.2)
Meglitinides	984 (7.6)	163 (0.7)	111 (2.0)	s	76 (0.1)	1,577 (2.5)	600 (4.6)	9 (0.2)
Insulin	2,308 (17.9)	3,487 (14.3)	724 (12.9)	115 (11.0)	12,288 (18.2)	8,369 (13.0)	3,294 (25.1)	429 (8.3)
Current antidiabetic drugs								
Metformin	7,302 (56.7)	11,749 (48.2)	2,063 (36.8)	456 (43.5)	32,259 (47.8)	40,963 (63.7)	7,017 (53.4)	1,317 (25.4)
Sulfonylureas	1,463 (11.4)	4,220 (17.3)	1,346 (24.0)	113 (10.8)	5,876 (8.7)	13,349 (20.8)	3,481 (26.5)	604 (11.6)
Thiazolidinediones	106 (0.8)	117 (0.5)	36 (0.6)	s	38 (0.1)	185 (0.3)	105 (0.8)	40 (0.8)
SGLT2 inhibitors	787 (6.1)	1,276 (5.2)	248 (4.4)	32 (3.1)	1,881 (2.8)	2,090 (3.2)	833 (6.3)	89 (1.7)
GLP-1 receptor agonists	44 (0.3)	80 (0.3)	s	s	0 (0.0)	15 (0.0)	13 (0.1)	8 (0.2)
Alpha-glucosidase inhibitors	14 (0.1)	73 (0.3)	28 (0.5)	0 (0.0)	132 (0.2)	126 (0.2)	35 (0.3)	s
Meglitinides	215 (1.7)	62 (0.3)	30 (0.5)	s	10 (0.0)	908 (1.4)	261 (2.0)	s
Insulin	604 (4.7)	992 (4.1)	167 (3.0)	29 (2.8)	3,425 (5.1)	3,096 (4.8)	1,011 (7.7)	154 (3.0)
No. of non-antidiabetic drugs^c^	7.1 **±** 4.8	7.6 ± 5.0	7.7 **±** 5.3	7.0 **±** 4.0	9.2 **±** 5.6	9.0 **±** 5.4	3.8 **±** 2.0	11.2 **±** 7.7
0-1	891 (6.9)	2,229 (9.1)	353 (6.3)	64 (6.1)	1,981 (2.9)	2,448 (3.8)	1,667 (12.7)	177 (3.4)
2-5	4,771 (37.1)	8,155 (33.4)	1,931 (34.5)	414 (39.5)	17,255 (25.6)	16,147 (25.1)	8,941 (68.0)	1,067 (20.6)
≥ 6	7,212 (56.0)	14,004 (57.4)	3,321 (59.3)	571 (54.4)	48,248 (71.5)	45,737 (71.1)	2,540 (19.3)	3,946 (76.0)
**Health care use** ^b^								
Inpatient hospitalizations								
0	11,245 (87.4)	18,514 (75.9)	4,939 (88.1)	959 (91.4)	53,671 (79.5)	47,923 (74.5)	9,616 (73.1)	3,512 (67.7)
1-2	1,501 (11.7)	5,113 (21.0)	629 (11.2)	80 (7.6)	12,333 (18.3)	14,567 (22.6)	3,051 (23.2)	1,317 (25.4)
≥ 3	128 (1.0)	761 (3.1)	37 (0.7)	10 (1.0)	1,480 (2.2)	1,842 (2.9)	481 (3.7)	361 (7.0)
Physician visits								
0	93 (0.7)	2,097 (8.6)	177 (3.2)	27 (2.6)	1,377 (2.0)	3,732 (5.8)	124 (0.9)	760 (14.6)
1-2	313 (2.4)	885 (3.6)	408 (7.3)	39 (3.7)	5,278 (7.8)	4,910 (7.6)	245 (1.9)	943 (18.2)
≥ 3	12,468 (96.9)	21,406 (87.8)	5,020 (89.6)	983 (93.7)	60,829 (90.1)	55,690 (86.6)	12,779 (97.2)	3,487 (67.2)

*Abbreviations*: *AB* Alberta, *BC* British Columbia, *CPRD* Clinical Practice Research Datalink, *DPP-4* Dipeptidyl peptidase-4, *GLP-1* Glucagon-like peptide-1, *MB* Manitoba, *NS* Nova Scotia, *ON* Ontario, *QC* Quebec, *SD* Standard deviation, *SGLT-2* Sodium-glucose co-transporter 2, *SK* Saskatchewan, *UK* United Kingdom

^a^Income quintile defined as low income households in BC and recipients of last-resort financial assistance in QC.

^b^Unless otherwise specified, medication use and healthcare use were assessed in the year prior to study cohort entry.

^c^Measured by drug class using site-specific approaches and assessed in the 365 days prior to and including study cohort entry.

^*^Data are presented as n (%) or mean ± SD. Values suppressed due to privacy restrictions are presented as s

**Table 3:** Characteristics of new users of SGLT-2 inhibitors by study site, 2016-2018

	AB (***n*** = 15,535)	BC (***n*** = 28,856)	MB (***n*** = 7,515)	NS (***n*** = 1,330)	O***N*** (***n*** = 56,389)	QC (***n*** = 42,805)	SK (***n*** = 10,799)	UK (***n*** = 3,493)
**Age (years)**	57.4 **±** 11.3	59.5 **±** 11.4	57.4 **±** 11.8	57.7 **±** 10.5	72.1 **±** 5.3	63.7 **±** 10.1	57.5 **±** 11.9	58.2 **±** 10.6
< 18	**–**	12 (0.0)	0 (0.0)	0 (0.0)	**–**	s	6 (0.1)	0 (0.0)
18-35	566 (3.6)	721 (2.5)	300 (4.0)	s	**–**	525 (1.2)	430 (4.0)	70 (2.0)
36-45	1,956 (12.6)	2,603 (9.0)	886 (11.8)	128 (9.6)	**–**	1,868 (4.4)	1,283 (11.9)	325 (9.3)
46-55	4,221 (27.2)	6,634 (23.0)	1,923 (25.6)	367 (27.6)	**–**	5,831 (13.6)	2,721 (25.2)	992 (28.4)
56-65	5,170 (33.3)	9,699 (33.6)	2,470 (32.9)	514 (38.7)	**–**	13,986 (32.7)	3,648 (33.8)	1,191 (34.1)
66-75	3,012 (19.4)	7,228 (25.0)	1,561 (20.8)	236 (17.7)	43,276 (76.7)	16,710 (39.0)	2,107 (19.5)	763 (21.8)
76-85	549 (3.5)	1,807 (6.3)	344 (4.6)	47 (3.5)	11,871 (21.1)	3,635 (8.5)	537 (5.0)	142 (4.1)
> 85	61 (0.4)	152 (0.5)	31 (0.4)	s	1,242 (2.2)	s	67 (0.6)	10 (0.3)
**Females**	6,241 (40.2)	11,369 (39.4)	3,308 (44.0)	533 (40.1)	22,964 (40.7)	17,427 (40.7)	4,504 (41.7)	1,466 (42.0)
**Income quintile** ^a^								
1^st^ (lowest)	3,802 (24.5)	7,075 (24.5)	1,598 (21.3)	204 (15.3)	12,662 (22.5)	5,605 (13.1)	2,256 (20.9)	1,232 (35.3)
2^nd^	3,398 (21.9)	**–**	1,630 (21.7)	238 (17.9)	12,467 (22.1)	**–**	2,045 (18.9)	506 (14.5)
3^rd^	2,764 (17.8)	**–**	1,517 (20.2)	227 (17.1)	11,681 (20.7)	**–**	2,420 (22.4)	667 (19.1)
4^th^	2,668 (17.2)	**–**	1,468 (19.5)	259 (19.5)	10,310 (18.3)	**–**	2,189 (20.3)	579 (16.6)
5^th^ (highest)	2,288 (14.7)	**–**	1,271 (16.9)	215 (16.2)	9,169 (16.3)	**–**	1,847 (17.1)	509 (14.6)
Missing	615 (4.0)	**–**	31 (0.4)	187 (14.1)	100 (0.2)	–	42 (0.4)	0 (0.0)
**Calendar year at cohort entry**								
2016	12,359 (79.6)	14,718 (51.0)	3,904 (51.9)	**–**	22,512 (39.9)	13,077 (30.6)	6,619 (61.3)	1,859 (53.2)
2017	3,176 (20.4)	9,155 (31.7)	2,829 (37.6)	295 (22.2)	27,819 (49.3)	20,109 (47.0)	2,552 (23.6)	1,634 (46.8)
2018	0 (0.0)	4,983 (17.3)	782 (10.4)	1,035 (77.8)	6,058 (10.7)	9,619 (22.5)	1,628 (15.1)	0 (0.0)
**Follow-up time (days)**	224 ± 132	509 ± 279	439 ± 244	111 ± 117	386 ± 219	409 ± 242	589 ± 304	325 ± 204
**Diabetes duration (years)**	9.7 ± 6.2	11.3 ± 7.3	11.6 ± 7.6	10.0 ± 6.3	14.6 ± 6.8	12.7 ± 6.2	10.7 ± 7.0	10.6 ± 6.7
< 1 year	1,031 (6.6)	2,743 (9.5)	408 (5.4)	98 (7.4)	1,379 (2.4)	1,085 (2.5)	694 (6.4)	120 (3.4)
1-4.9 years	3,176 (20.4)	3,855 (13.4)	1,249 (16.6)	260 (19.6)	4,021 (7.1)	4,572 (10.7)	1,984 (18.4)	595 (17.0)
5-10 years	4,267 (27.5)	6,582 (22.8)	1,821 (24.2)	337 (25.3)	9,306 (16.5)	9,027 (21.1)	2,813 (26.1)	1,049 (30.0)
> 10 years	7,061 (45.5)	15,676 (54.3)	4,037 (53.7)	635 (47.7)	41,683 (73.9)	28,121 (65.7)	5,308 (49.2)	1,729 (49.5)
**Use of medications** ^‡^								
No. of antidiabetic drugs	2.1 ± 1.1	1.8 ± 1.1	2.1 ± 0.9	2.0 ± 1.0	2.2 ± 1.0	2.5 ± 1.0	2.4 ± 1.1	2.1 ± 1.0
0	973 (6.3)	3,629 (12.6)	313 (4.2)	94 (7.1)	2,453 (4.4)	1,855 (4.3)	344 (3.2)	156 (4.5)
1	3,668 (23.6)	7,501 (26.0)	1,484 (19.7)	380 (28.6)	9,853 (17.5)	3,486 (8.1)	1,388 (12.9)	812 (23.2)
2	5,761 (37.1)	10,240 (35.5)	3,337 (44.4)	532 (40.0)	20,050 (35.6)	12,796 (29.9)	4,166 (38.6)	1,300 (37.2)
≥ 3	5,133 (33.0)	7,486 (25.9)	2,381 (31.7)	324 (24.4)	24,033 (42.6)	24,668 (57.6)	4,901 (45.4)	1,225 (35.1)
Prior antidiabetic drugs								
Metformin	13,229 (85.2)	22,986 (79.7)	6,610 (88.0)	1,091 (82.0)	47,869 (84.9)	37,555 (87.7)	9,338 (86.5)	3,115 (89.2)
Sulfonylureas	5,713 (36.8)	12,257 (42.5)	4,857 (64.6)	554 (41.7)	26,044 (46.2)	26,674 (62.3)	5,960 (55.2)	1,542 (44.1)
Thiazolidinediones	613 (4.0)	443 (1.5)	246 (3.3)	13 (1.0)	366 (0.6)	818 (1.9)	310 (2.9)	281 (8.0)
DPP-4 inhibitors	5,428 (34.9)	7,818 (27.1)	2,024 (26.9)	341 (25.6)	36,007 (63.9)	27,847 (65.1)	3,049 (28.2)	1,291 (37.0)
GLP-1 receptor agonists^§^	1,253 (8.1)	2,094 (7.3)	136 (1.8)	136 (10.2)	0 (0.0)	2,780 (6.5)	278 (2.6)	438 (12.5)
Alpha-glucosidase inhibitors	80 (0.5)	298 (1.0)	130 (1.7)	s	997 (1.8)	543 (1.3)	58 (0.5)	11 (0.3)
Meglitinides	1,215 (7.8)	170 (0.6)	120 (1.6)	s	49 (0.1)	1,194 (2.8)	417 (3.9)	9 (0.3)
Insulin	4,442 (28.6)	5,772 (20.0)	1,650 (22.0)	322 (24.2)	15,136 (26.8)	11,439 (26.7)	3,549 (32.9)	578 (16.5)
Current antidiabetic drugs								
Metformin	5,233 (33.7)	8,762 (30.4)	1,878 (25.0)	326 (24.5)	14,350 (25.4)	18,812 (43.9)	3,229 (29.9)	780 (22.3)
Sulfonylureas	1,201 (7.7)	3,265 (11.3)	1,279 (17.0)	103 (7.7)	5,519 (9.8)	10,274 (24.0)	1,494 (13.8)	279 (8.0)
Thiazolidinediones	146 (0.9)	89 (0.3)	37 (0.5)	s	48 (0.1)	215 (0.5)	41 (0.4)	34 (1.0)
DPP-4 inhibitors	1,858 (12.0)	2,503 (8.7)	499 (6.6)	82 (6.2)	10,798 (19.1)	13,221 (30.9)	983 (9.1)	184 (5.3)
GLP-1 receptor agonists	335 (2.2)	512 (1.8)	27 (0.4)	28 (2.1)	0 (0.0)	737 (1.7)	70 (0.7)	89 (2.5)
Alpha-glucosidase inhibitors	17 (0.1)	58 (0.2)	31 (0.4)	s	221 (0.4)	180 (0.4)	14 (0.1)	s
Meglitinides	224 (1.4)	35 (0.1)	14 (0.2)	0 (0.0)	s	376 (0.9)	98 (0.9)	s
Insulin	1,073 (6.9)	1,366 (4.7)	314 (4.2)	68 (5.1)	2,526 (4.5)	3,142 (7.3)	1,003 (9.3)	148 (4.2)
No. of non-antidiabetic drugs^c^	7.0 ± 4.5	7.1 ± 4.4	7.3 ± 4.9	8.0 ± 5.0	8.7 ± 5.0	9.0 ± 4.8	3.6 ± 2.0	11.3 ± 7.4
0-1	954 (6.1)	2,789 (9.7)	446 (5.9)	60 (4.5)	927 (1.6)	837 (2.0)	1,504 (13.9)	96 (2.7)
2-5	5,768 (37.1)	10,040 (34.8)	2,735 (36.4)	451 (33.9)	15,519 (27.5)	9,840 (23.0)	7,434 (68.8)	673 (19.3)
≥ 6	8,813 (56.7)	16,027 (55.5)	4,334 (57.7)	819 (61.6)	39,943 (70.8)	32,128 (75.1)	1,861 (17.2)	2,724 (78.0)
**Health care use** ^b^								
Inpatient hospitalizations								
0	14,038 (90.4)	23,048 (79.9)	6,831 (90.9)	1,179 (88.7)	49,967 (88.6)	35,390 (82.7)	8,088 (74.9)	2,645 (75.7)
1-2	1,419 (9.1)	5,339 (18.5)	654 (8.7)	144 (10.8)	6,049 (10.7)	6,905 (16.1)	2,466 (22.8)	737 (21.1)
≥3	78 (0.5)	469 (1.6)	30 (0.4)	7 (0.5)	373 (0.7)	510 (1.2)	245 (2.3)	111 (3.2)
Physician visits								
0	59 (0.4)	1,641 (5.7)	96 (1.3)	8 (0.6)	615 (1.1)	2,494 (5.8)	80 (0.7)	534 (15.3)
1-2	363 (2.3)	864 (3.0)	394 (5.2)	38 (2.9)	3,282 (5.8)	3,202 (7.5)	131 (1.2)	646 (18.5)
≥ 3	15,113 (97.3)	26,351 (91.3)	7,025 (93.5)	1,284 (96.5)	52,492 (93.1)	37,109 (86.7)	10,588 (98.1)	2,313 (66.2)

*Abbreviations*: *AB* Alberta, *BC* British Columbia, *CPRD* Clinical Practice Research Datalink, *DPP-4* Dipeptidyl peptidase-4, *GLP-1* Glucagon-like peptide-1, *MB* Manitoba, *NS* Nova Scotia, *ON* Ontario, *QC* Quebec, *SD* Standard deviation, *SGLT-2* Sodium-glucose co-transporter 2, *SK* Saskatchewan, *UK* United Kingdom

^a^Income quintile defined as low income households in BC and recipients of last-resort financial assistance in QC.

^b^Unless otherwise specified, medication use and healthcare use were assessed in the year prior to study cohort entry.

^c^Measured by drug class using site-specific approaches and assessed in the 365 days prior to and including study cohort entry.

^*^Data are presented as n (%) or mean ± SD. Values suppressed due to privacy restrictions are presented as s

**Table 4:** Characteristics of new users of GLP-1 receptor agonists by study site, 2016-2018

	AB (n = 4,665)	BC (n = 9,950)	MB (n = 696)	NS (n = 885)	QC (n = 8,817)	SK (n = 1,537)	UK (n = 1,169)
**Age (years)**	51.3 ± 11.3	54.9 ± 12.3	51.9 ± 12.2	53.2 ± 10.8	62.9 ± 10.2	53.2 ± 11.6	57.7 ± 10.9
< 18	**–**	16 (0.2)	s	s	s	s	0 (0.0)
18-35	485 (10.4)	705 (7.1)	66 (9.5)	53 (6.0)	140 (1.6)	110 (7.2)	s
36-45	989 (21.2)	1,470 (14.8)	146 (21.0)	143 (16.2)	433 (4.9)	278 (18.1)	116 (9.9)
46-55	1,523 (32.7)	2,700 (27.1)	200 (28.7)	299 (33.8)	1,229 (13.9)	445 (29.0)	344 (29.4)
56-65	1,248 (26.8)	3,008 (30.2)	182 (26.1)	284 (32.1)	3,055 (34.6)	484 (31.5)	383 (32.8)
66-75	388 (8.3)	1,747 (17.6)	90 (12.9)	90 (10.2)	3,329 (37.8)	194 (12.6)	249 (21.3)
76-85	s	290 (2.9)	10 (1.4)	12 (1.4)	593 (6.7)	s	40 (3.4)
> 85	s	14 (0.1)	s	s	s	s	s
**Females**	2,784 (59.7)	5,656 (56.8)	450 (64.7)	531 (60.0)	4,065 (46.1)	887 (57.7)	567 (48.5)
**Income quintile** ^a^							
1^st^ (lowest)	965 (20.7)	1,718 (17.3)	83 (11.9)	119 (13.5)	1,365 (15.5)	251 (16.3)	363 (31.1)
2^nd^	1,048 (22.5)	**–**	126 (18.1)	168 (19.0)	**–**	291 (18.9)	226 (19.3)
3^rd^	841 (18.0)	**–**	165 (23.7)	153 (17.3)	**–**	362 (23.6)	254 (21.7)
4^th^	868 (18.6)	**–**	156 (22.4)	148 (16.7)	**–**	359 (23.4)	186 (15.9)
5^th^ (highest)	764 (16.4)	**–**	166 (23.9)	141 (15.9)	**–**	266 (17.3)	140 (12.0)
Missing	179 (3.8)	**–**	0 (0.0)	156 (17.6)	–	8 (0.5)	0 (0.0)
**Calendar year at cohort entry**							
2016	3,678 (78.8)	3,622 (36.4)	292 (42.0)	**–**	2,493 (28.3)	764 (49.7)	631 (54.0)
2017	987 (21.2)	3,993 (40.1)	311 (44.7)	196 (22.2)	3,660 (41.5)	467 (30.4)	538 (46.0)
2018	0 (0.0)	2,335 (23.5)	93 (13.4)	689 (77.9)	2,664 (30.2)	306 (19.9)	0 (0.0)
**Follow-up time (days)**	223 ± 132	422 ± 263	390 ± 228	106 ± 73.4	373 ± 255	513 ± 303	309 ± 205
**Diabetes duration (years)**	7.1 ± 6.5	9.6 ± 8.0	9.2 ± 8.2	8.7 ± 6.9	13.7 ± 6.1	9.1 ± 7.5	11.9 ± 6.9
< 1 year	1,239 (26.6)	2,211 (22.2)	156 (22.4)	168 (19.0)	101 (1.1)	293 (19.1)	30 (2.6)
1-4.9 years	881 (18.9)	1,392 (14.0)	128 (18.4)	164 (18.5)	766 (8.7)	261 (17.0)	160 (13.7)
5-10 years	1,084 (23.2)	1,836 (18.5)	104 (14.9)	183 (20.7)	1,659 (18.8)	322 (21.0)	321 (27.5)
> 10 years	1,461 (31.3)	4,511 (45.3)	308 (44.3)	370 (41.8)	6,291 (71.4)	661 (43.0)	658 (56.3)
**Use of medications** ^‡^							
No. of antidiabetic drugs	1.6 ± 1.4	1.6 ± 1.4	2.0 ± 1.5	2.0 ± 1.0	3.2 ± 1.0	2.1 ± 1.5	2.3 ± 1.1
0	1,420 (30.4)	2,917 (29.3)	158 (22.7)	175 (19.8)	201 (2.3)	302 (19.7)	83 (7.1)
1	963 (20.6)	1,966 (19.8)	126 (18.1)	200 (22.6)	341 (3.9)	244 (15.9)	153 (13.1)
2	994 (21.3)	2,167 (21.8)	147 (21.1)	249 (28.1)	1,129 (12.8)	307 (20.0)	356 (30.5)
≥ 3	1,288 (27.6)	2,900 (29.1)	265 (38.1)	261 (29.5)	7,146 (81.0)	684 (44.5)	577 (49.4)
Prior antidiabetic drugs							
Metformin	2,856 (61.2)	6,023 (60.5)	463 (66.5)	612 (69.2)	7,995 (90.7)	996 (64.8)	972 (83.1)
Sulfonylureas	887 (19.0)	2,689 (27.0)	250 (35.9)	256 (28.9)	5,038 (57.1)	401 (26.1)	528 (45.2)
Thiazolidinediones	125 (2.7)	130 (1.3)	25 (3.6)	7 (0.8)	134 (1.5)	21 (1.4)	97 (8.3)
SGLT2 inhibitors	936 (20.1)	2,778 (27.9)	225 (32.3)	180 (20.3)	3,052 (34.6)	529 (34.4)	330 (28.2)
DPP-4 inhibitors	1,102 (23.6)	2,129 (21.4)	156 (22.4)	204 (23.1)	7,465 (84.7)	331 (21.5)	459 (39.3)
Alpha-glucosidase inhibitors	11 (0.2)	63 (0.6)	9 (1.3)	0 (0.0)	111 (1.3)	s	0 (0.0)
Meglitinides	231 (5.0)	51 (0.5)	15 (2.2)	s	341 (3.9)	45 (2.9)	s
Insulin	1,244 (26.7)	2,451 (24.6)	215 (30.9)	284 (32.1)	4,079 (46.3)	581 (37.8)	343 (29.3)
Current antidiabetic drugs							
Metformin	719 (15.4)	1,391 (14.0)	94 (13.5)	116 (13.1)	3,220 (36.5)	227 (14.8)	219 (18.7)
Sulfonylureas	133 (2.9)	438 (4.4)	41 (5.9)	19 (2.2)	1,380 (15.7)	64 (4.2)	96 (8.2)
Thiazolidinediones	18 (0.4)	21 (0.2)	s	0 (0.0)	18 (0.2)	s	12 (1.0)
SGLT2 inhibitors	276 (5.9)	654 (6.6)	41 (5.9)	24 (2.7)	953 (10.8)	129 (8.4)	56 (4.8)
DPP-4 inhibitors	95 (2.0)	176 (1.8)	8 (1.1)	12 (1.4)	346 (3.9)	39 (2.5)	28 (2.4)
Alpha-glucosidase inhibitors	0 (0.0)	7 (0.1)	s	0 (0.0)	24 (0.3)	0 (0.0)	s
Meglitinides	28 (0.6)	s	s	0 (0.0)	77 (0.9)	8 (0.5)	s
Insulin	318 (6.8)	623 (6.3)	40 (5.7)	66 (7.5)	1,207 (13.7)	168 (10.9)	103 (8.8)
No. of non-antidiabetic drugs^c^	7.6 ± 4.7	8.0 ± 4.8	8.1 ± 5.2	8.0 ± 5.0	10.7 ± 5.4	3.4 ± 2.1	13.7 ± 7.9
0-1	238 (5.1)	930 (9.3)	29 (4.2)	39 (4.4)	116 (1.3)	322 (21.0)	13 (1.1)
2-5	1,540 (33.0)	2,960 (29.7)	208 (29.9)	255 (28.8)	1,249 (14.2)	965 (62.8)	135 (11.5)
≥ 6	2,887 (61.9)	6,060 (60.9)	459 (65.9)	591 (66.8)	7,452 (84.5)	250 (16.3)	1,021 (87.3)
**Health care use** ^b^							
Inpatient hospitalizations							
0	4,255 (91.2)	7,865 (79.0)	631 (90.7)	821 (92.8)	7,009 (79.5)	1,187 (77.2)	824 (70.5)
1-2	385 (8.3)	1,900 (19.1)	65 (9.3)	s	1,634 (18.5)	318 (20.7)	280 (24.0)
≥ 3	25 (0.5)	185 (1.9)	0 (0.0)	s	174 (2.0)	32 (2.1)	65 (5.6)
Physician visits							
0	20 (0.4)	726 (7.3)	8 (1.1)	s	337 (3.8)	21 (1.4)	156 (13.3)
1-2	100 (2.1)	242 (2.4)	18 (2.6)	s	455 (5.2)	29 (1.9)	195 (16.7)
≥ 3	4,545 (97.4)	8,982 (90.3)	670 (96.3)	869 (98.2)	8,025 (91.0)	1,487 (96.8)	818 (70.0)

*Abbreviations*: *AB* Alberta, *BC* British Columbia, *CPRD* Clinical Practice Research Datalink, *DPP-4* Dipeptidyl peptidase-4, *GLP-1* Glucagon-like peptide-1, *MB* Manitoba, *NS* Nova Scotia, *QC* Quebec, *SD* Standard deviation, *SGLT-2* Sodium-glucose co-transporter 2, *SK* Saskatchewan, *UK* United Kingdom

^a^Income quintile defined as low income households in BC and recipients of last-resort financial assistance in QC.

^b^Unless otherwise specified, medication use and healthcare use were assessed in the year prior to study cohort entry.

^c^Measured by drug class using site-specific approaches and assessed in the 365 days prior to and including study cohort entry.

^*^Data are presented as n (%) or mean ± SD. Values suppressed due to privacy restrictions are presented as s. Data on the use of GLP-1 receptor agonists were not available in ON as these drugs are not covered by the provincial drug plan

**Fig. 1:**
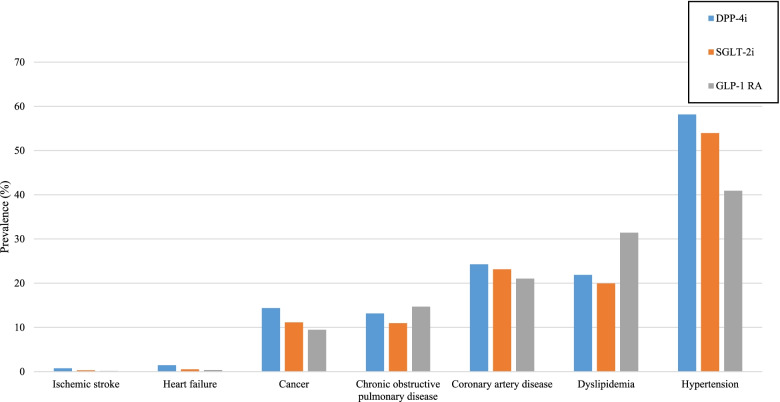
Prevalence of comorbidities at baseline in the Canadian sites. Abbreviations: DPP-4i: dipeptidyl peptidase-4 inhibitors; SGLT-2i: sodium-glucose co-transporter-2 inhibitors; GLP-1 RA: glucagon-like peptide-1 receptor agonists. * Assessed in the 3 years prior to cohort entry

Comorbidities of new users of DPP-4i, SGLT-2i, and GLP-1 RAs in Canada are presented in Figure 1. Hypertension was the most prevalent comorbidity among new users of DPP-4i (58%), SGLT-2i (54%), and GLP-1 RA (41%). The prevalence of coronary artery disease and dyslipidemia were between 20% and 31%, while the prevalence of cancer and chronic obstructive pulmonary disease ranged from 9% to 14% in each of the three study groups. New users of DPP-4i were more likely to have a history of coronary artery disease (24%) than new users of SGLT-2i (23%) or GLP-1 RA (21%). The prevalence of ischemic stroke ranged from 0.1% to 0.7%. The prevalence of heart failure was 1.3% for new-users of DPP-4i, 0.5% for new-users of SGLT-2i, and 0.3% for new-users of GLP-1 RA.

### Additional analyses

Characteristics of new users of DPP-4i, SGLT-2i, and GLP-1 RAs are presented for Canada and the UK in Tables S3, S4, and S5. The percentage of patients with less than one year of diabetes duration was greater among new users of GLP-1 RAs in Canada (15.7%) than in the UK (2.6%). In Canada, the majority of patients initiating one of the three study drugs had a diabetes duration of more than 10 years, whereas in the UK, the majority of DPP-4i and SGLT-2i users had a diabetes duration of less than 10 years. In addition, a greater percentage of patients using DPP-4i had diabetic nephropathy or other kidney diseases, both in Canada and the UK, as compared to users of SGLT-2i and GLP-1 RA.

Additional clinical, laboratory, and lifestyle data available for patients in the UK are presented in Table S6. Most patients using DPP-4i (52.3%), SGLT-2i (70.5%), and GLP-1 RAs (87.5%) had a BMI ≥30 kg/m^2^ at study cohort entry. Kidney function varied among the three groups, with 24.8% of DPP-4i users, 5.6% of SGLT-2i users, and 14.5% of GLP-1 RA users having an eGFR <60 mL/min/1.73m^2^. In all three groups, patients were mostly white, reported ever smoking, and had systolic and diastolic blood pressures under 140 and 90 mmHg, respectively.

Results for the sensitivity analysis comparing characteristics of new users of DPP-4i and SGLT-2i by reimbursement status in Manitoba are presented in Table S7. The distribution of user characteristics was similar between publicly and privately reimbursed drug claims.

## Discussion

The aim of this study was to describe the characteristics of new users of newer antidiabetic drugs in 7 Canadian provinces and in the UK between 2016 and 2018. Several characteristics differed amongst users of DPP-4i, SGLT-2i, and GLP-1 RA, namely age, sex, and the prevalence of certain comorbidities such as obesity and impaired renal function. Patients using DPP-4i had a higher prevalence of cardiovascular disease at baseline than those using SGLT-2i or GLP-1 RA.

Some use of DPP-4i, SGLT-2i, and GLP-1 RAs was not consistent with treatment guidelines in place during the study period. For example, between 16.2% and 26.9% of new users of all three classes of newer antidiabetic drugs had used insulin prior to treatment initiation with these drugs. However, American, Canadian, and UK guidelines generally recommend using insulin as a third line treatment, therefore only after failing treatment on second line therapies [1–3, 18] unless the patient has symptoms of hyperglycemia or evidence of metabolic decompensation at the time of type 2 diabetes diagnosis [2, 19]. Furthermore, a greater proportion of new users of DPP-4i had cardiovascular disease at baseline as compared to new users of SGLT-2i and GLP-1 RA, although SGLT-2i^2^ and GLP-1 RA [20] have a greater cardioprotective effect and are indicated for use in patients with type 2 diabetes at elevated cardiovascular risk [2]. In addition, a smaller proportion of patients using SGLT-2i had renal insufficiency (eGFR <60 mL/min/1.73m^2^) compared to users of DPP-4i and GLP-1 RA, although SGLT-2i can provide renal benefits in patients with type 2 diabetes. These findings suggest that patients who are most likely to benefit from SGLT-2i and GLP-1 RA may not be receiving these drugs, which has important implications for their health. However, varying formulary restrictions between provinces and changes in clinical practice guidelines throughout the study period may explain these discrepancies. Further research is needed to understand the observed discrepancies between treatment guidelines and what is implemented in routine clinical practice.

In Canada, health is a provincial and territorial responsibility. Consequently, the provinces and territories have the responsibility to manage their own drug plans, which results in important differences in eligibility and participation in drug plans across the country. As described in Table 1, there were important differences in the provincial formulary listings for SGLT-2i and GLP-1 RA, whereas the coverage of DPP-4i was relatively similar across provinces [2]. Formulary restrictions are a potential source of confounding and channeling in etiologic studies that may vary across provinces [21]. These variations may explain differences observed in the characteristics of new users of GLP-1 RAs across provinces; they were, on average, younger and more likely to have a shorter duration of diabetes and no prior use of antidiabetic medications in all provinces except Quebec, compared to users of SGLT-2i and DPP-4i. This suggests that GLP-1 RA, in particular liraglutide, may have been used for indications other than type 2 diabetes among these patients. Liraglutide is commercialized under two labels in Canada: Victoza®, indicated for type 2 diabetes, and Saxenda®, indicated for weight loss. Although we did not include Saxenda® in our analyses, it is possible that Victoza® was dispensed for the indication of weight loss to ensure insurance coverage.

Observed inter-provincial differences may also be the result of differences in data capture across provinces. In Alberta, British Columbia, Manitoba, and Nova Scotia, prescription drug data capture all dispensations, regardless of payer. However, drug dispensing data in Ontario and Quebec are only available for drugs that are covered by the provincial drug plans, while dispensations covered by the federal government are also captured in Saskatchewan. Given this potential source of heterogeneity, we conducted an analysis in Manitoba where we stratified new users by prescription payer. The results of this analysis suggest that the characteristics of patients who are covered by the public drug plan were similar to those of individuals who were not covered by the public plan. These potential sources of heterogeneity must be considered in the design of multi-jurisdictional studies and interpretation of pharmacoepidemiologic studies.

This study has several strengths. The use of administrative health databases from 7 Canadian sites and from the UK allowed us to comprehensively assess the use of newer 2^nd^ to 3^rd^ line antidiabetic drugs and the characteristics of patients initiating their use. Furthermore, the lifestyle, clinical and laboratory data available with the CPRD allowed us to assess patient characteristics that are not typically available in other administrative databases.

Our study also has some limitations. First, the dispensing of certain drugs may not have been measured due to availability of drug samples, which would not have been recorded in our databases. However, this should only affect a small proportion of all patients in our study and such dispensing is likely to be of short duration and followed by a recorded prescription. Second, while some patients may have entered the base cohort due to a dispensing of an antidiabetic drug for an indication other than type 2 diabetes, the three drug classes under investigation were largely prescribed for type 2 diabetes during the study period. Consequently, we do not anticipate many patients without type 2 diabetes being included in our study population. Third, we are unable to determine if the observed inter-provincial differences are because of true differences in use or differences in data capture across the provincial databases. In addition, we were unable to compare the utilization of studied drugs by molecule due to differences in formulary listings between jurisdictions, and differences in data capture. Fourth, the utilization of the studied drugs may have changed in these jurisdictions since the end of our data availability in 2018. Despite these limitations, our results provide an insightful picture of the patients receiving these newer antidiabetic drugs, which may inform policy makers and other decision makers on approaches to provide the most beneficial care.

## Conclusion

Although SGLT-2i, DPP-4i, and GLP-1 RAs are recommended as 2^nd^ or 3^rd^ line therapy for type 2 diabetes, important differences exist in the characteristics of users of these drugs. Our results also suggest that treatment for type 2 diabetes does not always follow recommended guidelines, where new users of DPP-4i had a higher prevalence of coronary artery disease than new users SGLT2i or GLP-1 RA. This study provides important insight into the complexity of treatment for type 2 diabetes. Further research is needed to better understand the consequences of differing drug plans across jurisdictions and differences in insurance systems across countries on medication utilization. In addition, future studies should examine the impact of changes in clinical evidence and guideline indications on prescribing trends.

## Supplementary Information

**Additional file 1.**

## Data Availability

This study was conducted by CNODES using administrative health data obtained through data-sharing agreements between its member research centres and their respective provincial data stewards. Data availability thus differs by site. **Alberta:** The authors cannot make the dataset used in this study available to other researchers due to their contractual arrangements with the provincial health ministry (Alberta Health), who is the data custodian. Researchers may make requests to obtain a similar dataset at https://absporu.ca/research-services/service-application/. **British Columbia:** The authors do not have permission to share data from this study. The data that support the findings of this study are available from Population Data BC (https://www.popdata.bc.ca/), but restrictions apply to the availability of these data, which were used under licence for the current study and so are not publicly available. **CPRD**: This study is based in part on data from the Clinical Practice Research Datalink obtained under license from the UK Medicines and Healthcare products Regulatory Agency. The data are provided by patients and collected by the UK National Health Service as part of their care and support. The interpretation and conclusions contained in this study are those of the author/s alone. Because electronic health records are classified as “sensitive data” by the UK Data Protection Act, information governance restrictions (to protect patient confidentiality) prevent data sharing via public deposition. Data are available with approval through the individual constituent entities controlling access to the data. Specifically, the primary care data can be requested via application to the Clinical Practice Research Datalink (https://www.cprd.com). **Manitoba:** Data used in this article was derived from administrative health and social data as a secondary source. The data was provided under specific data sharing agreements only for approved use at Manitoba Centre for Health Policy (MCHP). The original source data is not owned by the researchers or MCHP and as such cannot be provided to a public repository. The original data source and approval for use has been noted in the acknowledgments of the article. Where necessary, source data specific to this article or project may be reviewed at MCHP with the consent of the original data providers, along with the required privacy and ethical review bodies. **Nova Scotia**: Data in this study were obtained from Health Data Nova Scotia of Dalhousie University. These data can be acquired by researchers with an academic affiliation who submit a research protocol that is obtained by a Data Access Committee and Research Ethics Board. **Ontario**: The dataset from this study is held securely in coded form at ICES. While legal data sharing agreements between ICES and data providers (e.g., health organizations and government) prohibit ICES from making the dataset publicly available, access may be granted to those who meet pre-specified criteria for confidential access, available at www.ices.on.ca/DAS (email: das@ices.on.ca). **Quebec**: The data that support the findings of this study come from an analysis conducted by the Institut national d’excellence en santé et en services sociaux (INESSS). The results of this analysis are publicly available on INESSS’s Website. The original data used to produce this analysis are subject to confidentiality restrictions, and so are not publicly available under Quebec legislation. Authors may provide information concerning the original data upon reasonable request and with permission of INESSS. **Saskatchewan**: This study is based in-part on de-identified data provided by the Saskatchewan Ministry of Health. Restrictions apply to the availability of these data, which were used under license for the current study, and so are not publicly available. Data may be available from the authors upon reasonable request and with permission of the Saskatchewan Ministry of Health.

## Abbreviations

BMI: Body mass index
CNODES: Canadian Network for Observational Drug Effect Studies
CPRD: Clinical Practice Research Datalink
DPP-4i: Dipeptidyl Peptidase-4 inhibitors
eGFR: Estimated glomerular filtration rate
GLP-1 RA: Glucagon-like peptide-1 receptor agonists
HbA1c: Glycated hemoglobin
ICD: International Classification of Diseases
RCT: Randomized controlled trial
SGLT-2i: Sodium-glucose co-transporter-2 inhibitors
UK: United Kingdom
