# Supplementary material for: Characteristics of new users of recent antidiabetic drugs in Canada and the United Kingdom
Source: BMC Endocr Disord. 2022 Sep 29;22:241. doi: 10.1186/s12902-022-01140-1 (PMC9520836; doi:10.1186/s12902-022-01140-1)
Supplement: Supplementary file 1 — Additional file 1. [file 12902_2022_1140_MOESM1_ESM.docx]

**Characteristics of new users of recent antidiabetic drugs in Canada and the United Kingdom**

Vanessa C. Brunetti MSc,^1,2^ Audray St-Jean MSc, ^2^ Sophie Dell’Aniello MSc,^2^ Anat Fisher MD PhD,^3^ Oriana H.Y. Yu MD MSc,^2,4^ Shawn C. Bugden PharmD MSc,^5,6^ Jean-Marc Daigle MSc,^7^ Nianping Hu PhD,^8^ Silvia Alessi-Severini PhD,^5,9^ Baiju R. Shah MD PhD,^10,11^ Paul E. Ronksley PhD,^12^ Lisa M. Lix PhD,^13^ Pierre Ernst MD MSc,^2,14^ Kristian B. Filion PhD^1,2,14^ for the Canadian Network for Observational Drug Effect Studies (CNODES) Investigators^*^

Supplementary material

Table of Contents

[Table S1: Start and end dates of study periods at each site for our base cohort and prescription drug data coverage iii](#_Toc76465179)

[Table S2: Definitions of baseline characteristics iv](#_Toc76465180)

[Table S3: Baseline characteristics of new users of DPP-4 inhibitors, SGLT-2 inhibitors, and GLP-1 receptor agonists in Canada and the UK vii](#_Toc76465181)

[Table S4: Prior antidiabetic drug use of new users of DPP-4 inhibitors, SGLT-2 inhibitors, and GLP-1 receptor agonists in Canada and in the UK x](#_Toc76465182)

[Table S5: Baseline comorbidities in new users of DPP-4 inhibitors, SGLT-2 inhibitors, and GLP-1 receptor agonists in Canada and in the UK xi](#_Toc76465183)

[Table S6: Additional clinical, laboratory and lifestyle information on new users of DPP-4 inhibitors, SGLT-2 inhibitors and GLP-1 receptor agonists in the UK 2016-2018 xiii](#_Toc76465184)

[Table S7: Sensitivity analysis comparing characteristics of DPP-4 inhibitors and SGLT-2 inhibitors users by reimbursement status in Manitoba xiv](#_Toc76465185)

# Table S1: Start and end dates of study periods at each site for our base cohort and prescription drug data coverage.

| **Site** | **Start and end dates of study period** | **Prescription drug data coverage^*^** |
| --- | --- | --- |
| AB | January 1, 2008 – March 31, 2017 | All outpatient dispensations (≥18 years) |
| BC | January 1, 2006 – June 30, 2018 | All outpatient dispensations |
| MB | January 1, 2006 – March 31, 2018 | All outpatient dispensations |
| NS | November 1, 2016 – June 30, 2018 | All outpatient dispensations |
| ON | January 1, 2006 – March 31, 2018 | Outpatient dispensations covered by the provincial drug plan (≥65 years and social assistance recipient) |
| QC | January 1, 2006 – June 30, 2018 | Outpatient dispensations covered by the provincial drug plan (≥65 years, social assistance recipient and those with no other insurance) |
| SK | February 13, 2008 – June 30, 2018 | Outpatient dispensations covered by the provincial and federal drug plans |
| UK | January 1, 2006 – December 31, 2017 | Prescriptions (patients registered in a participating general practice) |

Abbreviations: AB: Alberta, BC: British Columbia, MB: Manitoba, NS: Nova Scotia, ON: Ontario, QC: Québec, SK: Saskatchewan, UK: United Kingdom

^*^Description of drug data available at each site, i.e., drug data type and groups covered.

# Table S2: Definitions of baseline characteristics.

| **Characteristic** | **Comment (if applicable)**^*^ |
| --- | --- |
| **Demographic** |  |
| Age | Defined at cohort entry date |
| Sex |  |
| Income quintile | Site-specific definition applied |
| **Diabetes duration** | Time since first diabetes diagnosis or treatment |
| **Use of medications** |  |
| No. of antidiabetic drugs | Measured by drug class using site-specific approaches and assessed in the 365 days prior to and including study cohort entry  Categorized as 0, 1, 2, or ≥3 |
| Metformin |  |
| Sulfonylureas |  |
| Thiazolidinediones |  |
| DPP-4 inhibitors |  |
| SGLT-2 inhibitors |  |
| GLP-1 receptor agonists |  |
| Alpha-glucosidase inhibitors |  |
| Meglitinides |  |
| Insulin |  |
| No. of non-antidiabetic drugs^†^ | Measured by drug class using site-specific approaches and assessed in the 365 days prior to and including study cohort entry  Categorized as 0-1, 2-5, or ≥6 |
| Angiotensin-converting enzyme inhibitors |  |
| Angiotensin receptor blockers |  |
| Beta-blockers |  |
| Calcium channel blockers |  |
| Loop diuretics |  |
| Thiazide diuretics |  |
| Other diuretics |  |
| Direct renin inhibitors |  |
| Aldosterone antagonists |  |
| Digitalis-like agents |  |
| Statins |  |
| Other lipid lowering therapy |  |
| Acetylsalicylic acid |  |
| Non-acetylsalicylic acid antiplatelets |  |
| Nonsteroidal anti-inflammatory drugs |  |
| Oral anticoagulants |  |
| Oral glucocorticoids |  |
| Atypical antipsychotics |  |
| **Comorbidities** |  |
| Myocardial infarction |  |
| Ischemic stroke |  |
| Diabetic ketoacidosis |  |
| Diabetic retinopathy |  |
| Diabetic neuropathy |  |
| Cancer | Excluding non-melanoma skin cancer |
| Diabetic nephropathy |  |
| Other kidney diseases |  |
| Dialysis | Defined using diagnosis and procedure codes |
| Alcohol-related disorders |  |
| Cirrhosis |  |
| Atrial fibrillation |  |
| Chronic obstructive pulmonary disease |  |
| Coronary artery disease |  |
| Dyslipidemia | Defined by a diagnosis of dyslipidemia (in the 3 years prior) or a prescription for a statin or other lipid lowering therapy (in the year prior) |
| Hypertension |  |
| Heart failure in the past 3 years |  |
| Venous thromboembolism |  |
| Peripheral arterial disease |  |
| Aortic aneurysm |  |
| Atherosclerosis |  |
| Cerebrovascular disease |  |
| Pyelonephritis |  |
| Cystitis |  |
| Stones or urinary tract obstruction |  |
| Urinary tract infection |  |
| Neurogenic bladder |  |
| Lower extremity amputation |  |
| **Healthcare use** |  |
| Number of inpatient hospitalizations | In the 365 days prior to and including study cohort entry  Categorized as 0, 1-2, or ≥3 |
| Number of physician visits | Included inpatient and outpatient visits in the 365 days prior to study cohort entry  Categorized as 0-2, 3-5, or ≥6 |
| **Additional CPRD covariates** |  |
| Body mass index (kg/m^2^) | Based on last measurement at any time before study cohort entry  Categorized as <30, ≥30, or missing |
| Smoking status | Based on last measurement at any time before study cohort entry  Categorized as never, ever, or missing |
| Race | Assessed at study cohort entry  Categorized as white, other, or missing |
| Blood pressure (mm Hg) | Based on last measurement at any time before study cohort entry  Categorized as DBP <90 and SBP <140, DBP ≥90 and SBP ≥140, or missing |
| eGFR (mL/min/1.73 m^2^) | Based on last measurement at any time before study cohort entry  Categorized as <60, ≥60 or missing |
| HbA1c level (%) | Based on last measurement at any time before study cohort entry  Categorized as ≤7, 7.1-8, >8 or missing |

Abbreviations: ATC, Anatomical Therapeutic Chemical; DBP, diastolic blood pressure; eGFR, estimated glomerular filtration rate; HbA1c, haemoglobin A1c; SBP, systolic blood pressure.

^*^Unless otherwise specified, medications and healthcare use were assessed in the year prior to study cohort entry. Comorbidities were ascertained from hospitalization or physician claims data in the three years prior to study cohort entry. Comorbidities were defined using ICD-9-CM for outpatient claims (except Ontario, which used ICD-8 codes, and CPRD, which used Read codes) and ICD-10-CA for hospitalization records. Procedures were defined using ICD-9-CM, Canadian Classification of Diagnostic, Therapeutic, and Surgical Procedures (CCP), Canadian Classification of Health Interventions (CCI) and site-specific procedure codes.

^†^In SK, the number of non-antidiabetic drug classes was defined using the list of medication covariates rather than ATC-defined classes due to unavailability of ATC codes.

# Table S3: Baseline characteristics of new users of DPP-4 inhibitors, SGLT-2 inhibitors, and GLP-1 receptor agonists in Canada and the UK.

| **Characteristic*** | **Canada** | | | **UK** | | |
| --- | --- | --- | --- | --- | --- | --- |
|  | **DPP-4 inhibitors** | **SGLT-2 inhibitors** | **GLP-1 receptor agonists** | **DPP-4 inhibitors** | **SGLT-2 inhibitors** | **GLP-1 receptor agonists** |
|  | **(n = 188,880)** | **(n = 163,229)** | **(n = 26,550)** | **(n = 5,190)** | **(n = 3,493)** | **(n = 1,169)** |
| **Age (years)** |  |  |  |  |  |  |
| < 18 | 25 (0.0) | 20 (0.0) | 27 (0.1) | s | s | s |
| 18-35 | 2,510 (1.3) | 2,575 (1.6) | 1,559 (5.9) | s | 70 (2.0) | 33 (2.8) |
| 36-45 | 7,735 (4.1) | 8,724 (5.3) | 3,459 (13.0) | 323 (6.2) | 325 (9.3) | 116 (9.9) |
| 46-55 | 18,894 (10.0) | 21,697 (13.3) | 6,396 (24.1) | 919 (17.7) | 992 (28.4) | 344 (29.4) |
| 56-65 | 35,369 (18.7) | 35,487 (21.7) | 8,261 (31.1) | 1,230 (23.7) | 1,191 (34.1) | 383 (32.8) |
| 66-75 | 75,492 (40.0) | 74,130 (45.4) | 5,838 (22.0) | 1,370 (26.4) | 763 (21.8) | 249 (21.3) |
| 76-85 | 37,607 (19.9) | 18,790 (11.5) | 939 (3.5) | 952 (18.3) | 142 (4.1) | 40 (3.4) |
| > 85 | 11,135 (5.9) | 1,806 (1.1) | 60 (0.2) | 314 (6.1) | s | s |
| **Females** | 83,733 (44.3) | 66,346 (40.7) | 14,373 (54.1) | 2,234 (43.0) | 1,466 (42.0) | 567 (48.5) |
| **Calendar year at cohort entry** |  |  |  |  |  |  |
| 2016 | 88,476 (46.8) | 73,189 (44.8) | 10,849 (40.9) | 3,041 (58.6) | 1,859 (53.2) | 631 (54.0) |
| 2017 | 72,671 (38.5) | 65,935 (40.4) | 9,614 (36.2) | 2,149 (41.4) | 1,634 (46.8) | 538 (46.0) |
| 2018 | 27,733 (14.7) | 24,105 (14.8) | 6,087 (22.9) | 0 (0.0) | 0 (0.0) | 0 (0.0) |
| **Diabetes duration** |  |  |  |  |  |  |
| < 1 year | 15,915 (8.4) | 7,438 (4.6) | 4,168 (15.7) | 306 (5.9) | 120 (3.4) | 30 (2.6) |
| 1-4.9 years | 26,255 (13.9) | 19,117 (11.7) | 3,592 (13.5) | 1,010 (19.5) | 595 (17.0) | 160 (13.7) |
| 5-10 years | 40,217 (21.3) | 34,153 (20.9) | 5,188 (19.5) | 1,405 (27.1) | 1,049 (30.0) | 321 (27.5) |
| > 10 years | 106,493 (56.4) | 105,521 (62.8) | 13,602 (51.2) | 2,469 (47.6) | 1,729 (49.5) | 658 (56.3) |
| **No. of non-antidiabetic drugs** |  |  |  |  |  |  |
| 0-1 | 12,466 (5.1) | 7,517 (4.6) | 1,674 (6.3) | 177 (3.4) | 96 (2.7) | 13 (1.1) |
| 2-5 | 57,614 (30.5) | 51,787 (31.7) | 7,177 (27.0) | 1,067 (20.6) | 673 (19.3) | 135 (11.5) |
| ≥ 6 | 121,633 (64.3) | 103,925 (63.7) | 17,699 (66.7) | 3,946 (76.0) | 2,724 (78.0) | 1,021 (87.3) |
| **Current use of antidiabetic drugs** |  |  |  |  |  |  |
| Metformin | 101,809 (53.9) | 52,590 (32.2) | 5,767 (21.7) | 1,317 (25.4) | 780 (22.3) | 219 (18.7) |
| Sulfonylureas | 29,848 (15.8) | 23,135 (14.2) | 2,075 (7.8) | 604 (11.6) | 279 (8.0) | 96 (8.2) |
| Thiazolidinediones | 590 (0.3) | 579 (0.4) | 63 (0.2) | 40 (0.8) | 34 (1.0) | 12 (1.0) |
| SGLT-2 inhibitors | 7,147 (3.8) | **–** | 2,077 (7.8) | 89 (1.7) | – | 56 (4.8) |
| DPP-4 inhibitors | **–** | 29,944 (18.3) | 676 (2.5) | – | 184 (5.3) | 28 (2.4) |
| GLP-1 receptor agonists | 158 (0.1) | 1,709 (1.0) | **–** | 8 (0.2) | 89 (2.5) | – |
| Alpha-glucosidase inhibitors | 408 (0.2) | 524 (0.3) | 34 (0.1) | s | s | s |
| Meglitinides | 1,489 (0.8) | 750 (0.5) | 119 (0.4) | s | s | s |
| Insulin | 9,324 (4.9) | 9,492 (5.8) | 2,422 (9.1) | 154 (3.0) | 148 (4.2) | 103 (8.8) |
| **Current use of non-antidiabetic drugs** |  |  |  |  |  |  |
| Angiotensin-converting enzyme inhibitors | 79,809 (42.3) | 74,023 (45.3) | 9,591 (36.1) | 2,273 (43.8) | 1,614 (46.2) | 581 (49.7) |
| Angiotensin receptor blockers | 59,042 (31.3) | 53,061 (32.5) | 7,774 (29.3) | 952 (18.3) | 598 (17.1) | 249 (21.3) |
| Beta-blockers | 60,200 (31.9) | 48,690 (29.8) | 6,409 (24.1) | 1,319 (25.4) | 695 (19.9) | 270 (23.1) |
| Calcium channel blockers | 63,641 (33.7) | 50,660 (31.0) | 6,647 (25.0) | 1,687 (32.5) | 1,081 (30.9) | 342 (29.3) |
| Loop diuretics | 24,764 (13.1) | 13,709 (8.4) | 2,517 (9.5) | 655 (12.6) | 216 (6.2) | 133 (11.4) |
| Thiazide diuretics | 46,360 (24.5) | 40,950 (25.1) | 6,182 (23.3) | 757 (14.6) | 477 (13.7) | 182 (15.6) |
| Other diuretics | 17,739 (9.4) | 16,080 (9.9) | 2,159 (8.1) | 200 (3.9) | 70 (2.0) | 50 (4.3) |
| Direct renin inhibitors | 84 (0.0) | 77 (0.0) | 19 (0.1) | 0 (0.0) | 0 (0.0) | 0 (0.0) |
| Aldosterone antagonists | 7,088 (3.8) | 5,045 (3.1) | 1,124 (4.2) | 186 (3.6) | 63 (1.8) | 46 (3.9) |
| Digitalis-like agents | 3,846 (2.0) | 2,139 (1.3) | 216 (0.8) | 138 (2.7) | 39 (1.1) | 21 (1.8) |
| Statins | 139,118 (73.7) | 126,296 (77.4) | 16,539 (62.3) | 3,869 (74.5) | 2,695 (77.2) | 914 (78.2) |
| Other lipid lowering therapy | 17,829 (9.4) | 19,050 (11.7) | 2,562 (9.6) | 183 (3.5) | 183 (5.2) | 60 (5.1) |
| Acetylsalicylic acid | 39,400 (20.9) | 30,762 (18.8) | 5,908 (22.3) | 1,329 (25.6) | 795 (22.8) | 289 (24.7) |
| Non-acetylsalicylic acid antiplatelets | 14,445 (7.6) | 11,820 (7.2) | 1,248 (4.7) | 434 (8.4) | 201 (5.8) | 65 (5.6) |
| Nonsteroidal anti-inflammatory drugs | 31,301 (16.6) | 30,817 (18.9) | 5,911 (22.3) | 648 (12.5) | 623 (17.8) | 201 (17.2) |
| Oral anticoagulants | 18,555 (9.8) | 11,518 (7.1) | 1,520 (5.7) | 438 (8.4) | 130 (3.7) | 67 (5.7) |
| Oral glucocorticoids | 15,581 (8.2) | 10,284 (6.3) | 2,319 (8.7) | 431 (8.3) | 271 (7.8) | 103 (8.8) |
| Atypical antipsychotics | 10,935 (5.8) | 7,142 (4.4) | 1,695 (6.4) | 103 (2.0) | 63 (1.8) | 36 (3.1) |

Abbreviations: DPP-4, dipeptidyl peptidase-4; GLP-1, glucagon-like peptide-1; SD, standard deviation; SGLT-2, sodium-glucose co-transporter 2; UK: United Kingdom

^*^Data are presented as n (%) or mean ± SD. Values suppressed due to privacy restrictions are presented as s.

# Table S4: Prior antidiabetic drug use of new users of DPP-4 inhibitors, SGLT-2 inhibitors, and GLP-1 receptor agonists in Canada and in the UK.^*^

| **Medications** | **Canada** | | | **UK** | | |
| --- | --- | --- | --- | --- | --- | --- |
|  | **DPP-4 inhibitors** | **SGLT-2 inhibitors** | **GLP-1 receptor agonists** | **DPP-4 inhibitors** | **SGLT-2 inhibitors** | **GLP-1 receptor agonists** |
|  | **(n = 188,880)** | **(n = 163,229)** | **(n = 26,550)** | **(n = 5,190)** | **(n = 3,493)** | **(n = 1,169)** |
| **No. of antidiabetic drugs, n (%)** |  |  |  |  |  |  |
| 0 | 27,031 (14.3) | 9,661 (5.9) | 5,173 (19.5) | 728 (14.0) | 156 (4.5) | 83 (7.1) |
| 1 | 69,425 (36.8) | 27,760 (17.0) | 3,840 (14.5) | 2,286 (44.0) | 812 (23.2) | 153 (13.1) |
| 2 | 69,463 (36.8) | 56,882 (34.8) | 4,993 (18.8) | 1,757 (33.9) | 1,300 (37.2) | 356 (30.5) |
| ≥ 3 | 22,961 (12.2) | 68,926 (42.2) | 12,544 (47.2) | 419 (8.1) | 1,225 (35.1) | 577 (49.4) |
| **Prior antidiabetic drugs, n (%)** |  |  |  |  |  |  |
| Metformin | 142,704 (75.6) | 138,678 (85.0) | 18,945 (71.4) | 4,042 (77.9) | 3,115 (89.2) | 972 (83.1) |
| Sulfonylureas | 77,441 (41.0) | 82,059 (50.3) | 9,521 (35.9) | 1,900 (36.6) | 1,542 (44.1) | 528 (45.2) |
| Thiazolidinediones | 2,767 (1.5) | 2,809 (1.7) | 442 (1.7) | 261 (5.0) | 281 (8.0) | 97 (8.3) |
| SGLT-2 inhibitors | 15,032 (8.0) | - | 7,700 (29.0) | 349 (6.7) | - | 330 (28.2) |
| DPP-4 inhibitors | - | 82,514 (50.6) | 11,387 (42.9) | - | 1,291 (37.0) | 459 (39.3) |
| GLP-1 receptor agonists | 1,213 (0.6) | 6,677 (4.1) | - | 116 (2.2) | 438 (12.5) | - |
| Alpha-glucosidase inhibitors | 1,168 (0.6) | 2,109 (1.3) | 197 (0.7) | 12 (0.2) | 11 (0.3) | 0 (0.0) |
| Meglitinides | 3,514 (1.9) | 3,168 (1.9) | 686 (2.6) | 9 (0.2) | 9 (0.3) | s |
| Insulin | 30,585 (16.2) | 42,310 (25.9) | 8,854 (33.3) | 429 (8.3) | 578 (16.5) | 343 (29.3) |

Abbreviations: DPP-4, dipeptidyl peptidase-4; GLP-1, glucagon-like peptide-1; SD, standard deviation; SGLT-2, sodium-glucose co-transporter 2; UK: United Kingdom

^*^Data are presented as n (%) or mean ± SD. Values suppressed due to privacy restrictions are presented as s.

# Table S5: Baseline comorbidities in new users of DPP-4 inhibitors, SGLT-2 inhibitors, and GLP-1 receptor agonists in Canada and in the UK.^*^

|  | **Canada** | | | **UK** | | |
| --- | --- | --- | --- | --- | --- | --- |
| **Comorbidities** | **DPP-4 inhibitors**  **(n = 188,880)** | **SGLT-2 inhibitors**  **(n = 163,229)** | **GLP-1 receptor agonists**  **(n = 26,550)** | **DPP-4 inhibitors**  **(n = 5,190)** | **SGLT-2 inhibitors**  **(n = 3,493)** | **GLP-1 receptor agonists**  **(n = 1,169)** |
| Myocardial infarction | 2,021 (1.1) | 1,561 (1.0) | 133 (0.5) | 45 (0.9) | 18 (0.5) | s |
| Ischemic stroke | 1,375 (0.7) | 486 (0.3) | 38 (0.1) | 44 (0.8) | 16 (0.5) | s |
| Diabetic ketoacidosis | 1,410 (0.7) | 881 (0.5) | 235 (0.9) | 48 (0.9) | 24 (0.7) | 20 (1.7) |
| Diabetic retinopathy | 4,946 (2.6) | 4,499 (2.8) | 1,126 (4.2) | 1,438 (27.7) | 882 (25.3) | 351 (30.0) |
| Diabetic neuropathy | 4,107 (2.2) | 2,275 (1.4) | 685 (2.6) | 901 (17.4) | 673 (19.3) | 258 (22.1) |
| Cancer | 26,279 (13.9) | 17,806 (10.9) | 2,404 (9.1) | 913 (17.6) | 427 (12.2) | 159 (13.6) |
| Diabetic nephropathy | 10,136 (5.4) | 4,113 (2.5) | 852 (3.2) | 65 (1.3) | 18 (0.5) | 9 (0.8) |
| Other kidney diseases | 23,129 (12.2) | 8,266 (5.1) | 2,105 (7.9) | 1,212 (23.4) | 302 (8.6) | 169 (14.5) |
| Dialysis | 1,606 (0.9) | 242 (0.1) | 67 (0.3) | 55 (1.1) | 7 (0.2) | 9 (0.8) |
| Alcohol-related disorders | 4,724 (2.5) | 2,722 (1.7) | 444 (1.7) | 370 (7.1) | 280 (8.0) | 88 (7.5) |
| Cirrhosis | 3,480 (1.8) | 2,879 (1.8) | 710 (2.7) | 53 (1.0) | 36 (1.0) | 9 (0.8) |
| Atrial fibrillation | 12,312 (6.5) | 6,142 (3.8) | 1,094 (4.1) | 529 (10.2) | 172 (4.9) | 71 (6.1) |
| Chronic obstructive pulmonary disease | 23,310 (12.3) | 17,095 (10.5) | 3,444 (13.0) | 705 (13.6) | 383 (11.0) | 172 (14.7) |
| Coronary artery disease | 43,936 (23.3) | 36,871 (22.6) | 4,912 (18.5) | 1,595 (30.7) | 819 (23.4) | 278 (23.8) |
| Dyslipidemia | 38,532 (20.4) | 31,208 (19.1) | 6,353 (23.9) | 2,254 (43.4) | 1,316 (37.7) | 457 (39.1) |
| Hypertension | 102,355 (54.2) | 84,221 (51.6) | 12,875 (48.5) | 3,934 (75.8) | 2,510 (71.9) | 914 (78.2) |
| Heart failure in the past 3 years | 18,529 (9.8) | 10,230 (6.3) | 1,438 (5.4) | 427 (8.2) | 112 (3.2) | 68 (5.8) |
| Venous thromboembolism | 3,948 (2.1) | 2,278 (1.4) | 606 (2.3) | 358 (6.9) | 209 (6.0) | 98 (8.4) |
| Peripheral arterial disease | 5,584 (3.0) | 3,916 (2.4) | 341 (1.3) | 516 (9.9) | 279 (8.0) | 110 (9.4) |
| Aortic aneurysm | 2,058 (1.1) | 1,289 (0.8) | 138 (0.5) | 76 (1.5) | 18 (0.5) | 6 (0.5) |
| Atherosclerosis | 6,032 (3.2) | 3,616 (2.2) | 685 (2.6) | 91 (1.8) | 18 (0.5) | 8 (0.7) |
| Cerebrovascular disease | 13,719 (7.3) | 7,969 (4.9) | 1,005 (3.8) | 578 (11.1) | 189 (5.4) | 60 (5.1) |
| Pyelonephritis | 1,908 (1.0) | 916 (0.6) | 133 (0.5) | 84 (1.6) | 58 (1.7) | 35 (3.0) |
| Cystitis | 15,744 (8.3) | 11,439 (7.0) | 2,433 (9.2) | 585 (11.3) | 419 (12.0) | 166 (14.2) |
| Stones or urinary tract obstruction | 8,778 (4.6) | 6,659 (4.1) | 1,173 (4.4) | 277 (5.3) | 172 (4.9) | 64 (5.5) |
| Urinary tract infection | 12,343 (6.5) | 6,251 (3.8) | 1,542 (5.8) | 1,130 (21.8) | 676 (19.4) | 280 (24.0) |
| Neurogenic bladder | 319 (0.2) | 117 (0.1) | 52 (0.2) | 13 (0.3) | 14 (0.4) | 6 (0.5) |
| Lower extremity amputation | 1,000 (0.5) | 540 (0.3) | 98 (0.4) | 70 (1.3) | 20 (0.6) | 16 (1.4) |

Abbreviations: DPP-4: dipeptidyl-peptidase 4; SGLT-2: sodium-glucose co-transporter 2, GLP-1: glucagon-like peptide 1; UK: United Kingdom

* Data are presented as n (%) or mean ± SD. Values suppressed due to privacy restrictions are presented as s.

# Table S6: Additional clinical, laboratory and lifestyle information on new users of DPP-4 inhibitors, SGLT-2 inhibitors, and GLP-1 receptor agonists in the UK 2016-2018.*

| **Characteristic** | **DPP-4 inhibitors**  **(n = 5,190)** | **SGLT-2 inhibitors**  **(n = 3,493)** | **GLP-1 receptor agonists**  **(n = 1,169)** |
| --- | --- | --- | --- |
| Body Mass Index (kg/m^2^) |  |  |  |
| ˂ 30 | 2,305 (44.4) | 995 (28.5) | 126 (10.8) |
| ≥ 30 | 2,712 (52.3) | 2,463 (70.5) | 1,023 (87.5) |
| Unknown | 173 (3.3) | 35 (1.0) | 20 (1.7) |
| Smoking |  |  |  |
| Never | 2,090 (40.3) | 1,410 (40.4) | 445 (38.1) |
| Ever | 2,952 (56.9) | 2,049 (58.7) | 708 (60.6) |
| Unknown | 148 (2.9) | 34 (1.0) | 16 (1.4) |
| Ethnicity |  |  |  |
| White | 3,667 (70.7) | 2,426 (69.5) | 932 (79.7) |
| Other | 655 (12.6) | 422 (12.1) | 89 (7.6) |
| Unknown | 868 (16.7) | 645 (18.5) | 148 (12.7) |
| Blood pressure level (mm Hg) |  |  |  |
| DBP <90 and SBP <140 | 3,308 (63.7) | 2,229 (63.8) | 743 (63.6) |
| DBP ≥90 or SBP ≥140 | 1,672 (32.2) | 1,223 (35.0) | 384 (32.8) |
| Unknown | 210 (4.0) | 41 (1.2) | 42 (3.6) |
| eGFR **(**mL/min/1.73m^2^) |  |  |  |
| < 60 | 1,286 (24.8) | 194 (5.6) | 170 (14.5) |
| ≥ 60 | 3,538 (68.2) | 3,207 (91.8) | 939 (80.3) |
| Unknown | 366 (7.1) | 92 (2.6) | 60 (5.1) |
| HbA1c level (%) |  |  |  |
| ≤ 7 | 465 (9.0) | 135 (3.9) | 62 (5.3) |
| 7.1-8 | 1,337 (25.8) | 635 (18.2) | 140 (12.0) |
| > 8 | 3,003 (57.9) | 2,627 (75.2) | 906 (77.5) |
| Unknown | 385 (7.4) | 96 (2.7) | 61 (5.2) |

Abbreviations: DPP-4: dipeptidyl-peptidase 4; SGLT-2: sodium-glucose co-transporter 2, GLP-1: glucagon-like peptide 1; UK: United Kingdom; DBP: diastolic blood pressure; SBP: systolic blood pressure; GFR: glomerular filtration rate; HbA1c: hemoglobin A1c

*Data presented as n(%).

# Table S7: Sensitivity analysis comparing characteristics of DPP-4 inhibitors and SGLT-2 inhibitors users by reimbursement status in Manitoba.^*^

| **Characteristics**^†^ | **DDP-4 inhibitors** | | **SGLT2 inhibitors** | |
| --- | --- | --- | --- | --- |
|  | **Public**  **(n = 1,546)** | **Private**  **(n = 4,059)** | **Public**  **(n = 1,525)** | **Private**  **(n = 5,990)** |
| **Age (years)** | 62 (13.2) | 57.2 (13.4) | 59.8 (12.0) | 56.8 (11.7) |
| < 18 | 0 (0.0) | s | 0 (0.0) | 0 (0.0) |
| 18-35 | 41 (2.7) | 234 (5.8) | 48 (3.1) | 252 (4.2) |
| 36-45 | 131 (8.5) | 544 (13.4) | 139 (9.1) | 747 (12.5) |
| 46-55 | 300 (19.4) | 1,025 (25.3) | 330 (21.6) | 1,593 (26.6) |
| 56-65 | 452 (29.2) | 1,153 (28.4) | 521 (34.2) | 1,949 (32.5) |
| 66-75 | 373 (24.1) | 753 (18.6) | 354 (23.2) | 1,207 (20.2) |
| 76-85 | 200 (12.9) | 280 (6.9) | 119 (7.8) | 225 (3.8) |
| > 85 | 49 (3.2) | s | 14 (0.9) | 17 (0.3) |
| **Females** | 753 (48.7) | 1,933 (47.6) | 647 (42.4) | 2661 (44.4) |
| **Income quintile** |  |  |  |  |
| 1st (Lowest) | 325 (21.0) | 1,178 (29.0) | 310 (20.3) | 1,288 (21.5) |
| 2nd | 336 (21.7) | 929 (22.9) | 339 (22.2) | 1,291 (21.6) |
| 3rd | 343 (22.2) | 723 (17.8) | 334 (21.9) | 1,183 (19.7) |
| 4th | 284 (18.4) | 661 (16.3) | 298 (19.5) | 1,170 (19.5) |
| 5th (Highest) | 236 (15.3) | 545 (13.4) | 230 (15.1) | 1,041 (17.4) |
| Missing | 22 (1.4) | 23 (0.6) | 14 (0.9) | 17 (0.3) |
| **Calendar year of new user cohort entry date** |  |  |  |  |
| 2016 | 694 (44.9) | 1,872 (46.1) | 386 (25.3) | 3,518 (58.7) |
| 2017 | 676 (43.7) | 1,740 (42.9) | 855 (56.1) | 1,974 (33.0) |
| 2018 | 176 (11.4) | 447 (11.0) | 284 (18.6) | 498 (8.3) |
| **Follow-up time** (days), mean (SD) | 407.7 (237.8) | 407.5 (238.0) | 309.9 (218.0) | 471.3 (239.9) |
| **Diabetes duration (years), mean (SD)** | 11.8 (7.8) | 11.2 (8.0) | 11.5 (7.2) | 11.7 (7.7) |
| < 1 year | 80 (5.2) | 332 (8.2) | 51 (3.3) | 357 (6.0) |
| 1-4.9 years | 253 (16.4) | 751 (18.5) | 257 (16.9) | 992 (16.6) |
| 5-10 years | 383 (24.8) | 943 (23.2) | 405 (26.6) | 1,416 (23.6) |
| > 10 years | 830 (53.7) | 2,033 (50.1) | 812 (53.2) | 3,225 (53.8) |
| **Use of medications** |  |  |  |  |
| No. of antidiabetic drugs, mean (SD) | 2 (0.8) | 1.8 (0.9) | 2.2 (0.8) | 2.1 (1.0) |
| 0 | 46 (3.0) | 292 (7.2) | 24 (1.6) | 289 (4.8) |
| 1 | 330 (21.3) | 897 (22.1) | 238 (15.6) | 1,246 (20.8) |
| 2 | 854 (55.2) | 2,132 (52.5) | 804 (52.7) | 2,533 (42.3) |
| 3+ | 316 (20.4) | 738 (18.2) | 459 (30.1) | 1,922 (32.1) |
| Prior antidiabetic drugs |  |  |  |  |
| Metformin | 1,351 (87.4) | 3,379 (83.2) | 1,391 (91.2) | 5,219 (87.1) |
| Sulfonylureas | 1,185 (76.6) | 2,740 (67.5) | 1,211 (79.4) | 3,646 (60.9) |
| Thiazolidinediones | 50 (3.2) | 90 (2.2) | 68 (4.5) | 178 (3.0) |
| SGLT2 inhibitors | 288 (18.6) | 478 (11.8) | 0 (0.0) | 0 (0.0) |
| DPP-4 inhibitors | 0 (0.0) | 0 (0.0) | 435 (28.5) | 1,589 (26.5) |
| GLP-1 receptor agonists | 11 (0.7) | 15 (0.4) | 19 (1.2) | 117 (2.0) |
| Alpha-glucosidase inhibitors | 43 (2.8) | 46 (1.1) | 27 (1.8) | 103 (1.7) |
| Meglitinides | 29 (1.9) | 82 (2.0) | 25 (1.6) | 95 (1.6) |
| Insulin | 90 (5.8) | 634 (15.6) | 115 (7.5) | 1,535 (25.6) |
| Current antidiabetic drugs |  |  |  |  |
| Metformin | 410 (26.5) | 1,653 (40.7) | 308 (20.2) | 1,570 (26.2) |
| Sulfonylureas | 298 (19.3) | 1,048 (25.8) | 262 (17.2) | 1,017 (17.0) |
| Thiazolidinediones | 17 (1.1) | 19 (0.5) | 10 (0.7) | 27 (0.5) |
| SGLT2 inhibitors | 92 (6.0) | 156 (3.8) | 1,525 (100.0) | 5,990 (100.0) |
| DPP-4 inhibitors | 1,546 (100.0) | 4,059 (100.0) | 96 (6.3) | 403 (6.7) |
| GLP-1 receptor agonists | 0 (0.0) | s | s | 24 (0.4) |
| Alpha-glucosidase inhibitors | 12 (0.8) | 16 (0.4) | 7 (0.5) | 24 (0.4) |
| Meglitinides | 8 (0.5) | 22 (0.5) | s | 11 (0.2) |
| Insulin | 13 (0.8) | 154 (3.8) | 9 (0.6) | 305 (5.1) |
| Number of unique non-antidiabetic medications, mean (SD) | 7.6 (4.9) | 7.7 (5.4) | 7.4 (4.6) | 7.3 (5.0) |
| 0-1 | 70 (4.5) | 283 (7.0) | 70 (4.6) | 376 (6.3) |
| 2-5 | 543 (35.1) | 1,388 (34.2) | 535 (35.1) | 2,200 (36.7) |
| 6+ | 933 (60.3) | 2,388 (58.8) | 920 (60.3) | 3,414 (57.0) |
| **Health care use** |  |  |  |  |
| Inpatient hospitalizations |  |  |  |  |
| 0 | 1,363 (88.2) | 3,576 (88.1) | 1,375 (90.2) | 5,456 (91.1) |
| 1-2 | 175 (11.3) | 454 (11.2) | 140 (9.2) | 514 (8.6) |
| 3+ | 8 (0.5) | 29 (0.7) | 10 (0.7) | 20 (0.3) |
| Number of physician visits |  |  |  |  |
| 0 | 11 (0.7) | 166 (4.1) | 13 (0.9) | 83 (1.4) |
| 1-2 | 49 (3.2) | 359 (8.8) | 57 (3.7) | 337 (5.6) |
| 3+ | 1,486 (96.1) | 3,534 (87.1) | 1,455 (95.4) | 5,570 (93.0) |

Abbreviations: DPP-4: dipeptidyl peptidase-4, SGLT-2: sodium-glucose co-transporter 2, GLP-1: glucagon-like peptide 1

*Reimbursement status defined as publicly vs. privately reimbursed claims.

^†^Data presented as n (%) or mean (SD). Values suppressed due to privacy restrictions are presented as s.
